# Polymers for Improving the *In Vivo* Transduction Efficiency of AAV2 Vectors

**DOI:** 10.1371/journal.pone.0015576

**Published:** 2010-12-28

**Authors:** Gilles Moulay, Sylvie Boutin, Carole Masurier, Daniel Scherman, Antoine Kichler

**Affiliations:** 1 Research Department, Genethon, Evry, France; 2 UMR 8151 CNRS-U1022 INSERM, Université René Descartes, Chimie Paristech, Paris, France; Universidade Federal do Rio de Janeiro, Brazil

## Abstract

**Background:**

Adeno-associated virus has attracted great attention as vehicle for body-wide gene delivery. However, for the successful treatment of a disease such as Duchenne muscular dystrophy infusion of very large amounts of vectors is required. This not only raises questions about the technical feasibility of the large scale production but also about the overall safety of the approach. One way to overcome these problems would be to find strategies able to increase the *in vivo* efficiency.

**Methodology:**

Here, we investigated whether polymers can act as adjuvants to increase the *in vivo* efficiency of AAV2. Our strategy consisted in the pre-injection of polymers before intravenous administration of mice with AAV2 encoding a murine secreted alkaline phosphatase (mSeAP). The transgene expression, vector biodistribution and tissue transduction were studied by quantification of the mSeAP protein and real time PCR. The injection of polyinosinic acid and polylysine resulted in an increase of plasmatic mSeAP of 2- and 12-fold, respectively. Interestingly, polyinosinic acid pre-injection significantly reduced the neutralizing antibody titer raised against AAV2.

**Conclusions:**

Our results show that the pre-injection of polymers can improve the overall transduction efficiency of systemically administered AAV2 and reduce the humoral response against the capsid proteins.

## Introduction

Adeno-associated virus mediated gene transfer has a great potential due to its capacity to transduce a wide spectrum of both dividing and non-dividing cell types. For example, widespread transduction of skeletal muscles in adult mice and hamster after a single intravenous administration of recombinant adeno-associated virus has been described [1], [2]. These results open new hopes for the treatment by gene therapy of neuromuscular diseases such as Duchenne muscular dystrophy (DMD). However, the broad tropism of the AAV vectors presents also a drawback since it precludes specific *in vivo* targeting. Another consequence when considering a gene therapy approach for a disorder such as DMD is that because a majority of the diseased cells have to be transduced in order to have a therapeutic benefit, whole-body AAV transduction of muscle requires the injection of very large amounts of vectors. For human application, this raises not only safety concerns but it also represents a hurdle at the level of large scale production of rAAVs. One way to overcome both problems would be to find strategies able to increase the transduction efficiency *in vivo*, without increasing the safety risks. Attempts to increase the transduction efficiency or to modify the tropism have been done by using genetic capsid modification approaches. These include for example site-directed mutagenesis of surface-exposed tyrosine residues [3]–[5] and incorporation of targeting peptides selected by phage display on the surface of AAV capsids [6], [7]. Alternatively, to engineer gene vectors that target a given tissue, random libraries of adeno-associated virus (AAV) can be generated by shuffling the capsid genes [8].

A few non-genetic strategies for enhancing the efficiency of recombinant AAV have also been developed. Walters and co-workers for example have shown that incorporation of AAV2 in a calcium phosphate co-precipitate improves gene transfer to airway epithelia after intranasal instillation [9]. More recently, it was shown that addition of cationic lipids to AAV2/9 increased the transduction efficiency of the lung [10]. Covalent modification of the capsid by either polyethyleneglycol chains [11] or by HPMA copolymers have also been evaluated [12].

In the present work, we investigated two original non-genetic strategies to enhance the *in vivo* efficiency of AAV. For our study, we focused on AAV2 because among the currently used AAV serotypes, it is the most studied and best characterized serotype, as well as the most used in clinical trials. The strategies that we have studied here consisted in the pre-injection of cationic or anionic polymers a few minutes before intravenous infusion of the AAV2 viral particles. The anionic polymer which we focused on was polyinosinic acid (pI). This compound was chosen since it has been previously reported to bind scavenger receptors and because it has been shown that an injection of pI prior to adenovirus administration results in enhanced transgene expression [13]. It was therefore of interest to test whether the pre-injection of pI could prevent infection by AAV2 of Kupffer cells or other antigen presenting cells, and thus, not only enhance the transduction of other tissues but also reduce the humoral immune response against AAV2 capsid proteins.

We have also evaluated the effect of pre- or co-injection of a series of cationic polymers, namely polylysines (pLys) with different degrees of polymerization (dp). The rationale was that AAV2 uses cell membrane-associated heparan sulfate proteoglycan (HSPG) as its primary binding receptor [14], [15]. This binding is driven by electrostatic interaction between negative HSPG and five positively charged amino acids [16]. Notably, the insertion of negatively charged peptides in this HSPG binding motif was shown to ablate HSPG binding, whereas positively charged peptides could restore the interactions [17]. Since polylysine is able to bind through electrostatic interactions to HSPG [18], we hypothesized that this polymer could alter the AAV biodistribution and transduction efficiency.

The experiments were conducted using a murine secreted alkaline phosphatase (mSeAP), whose production can be quantified in blood samples as well as on tissue sections. Vector biodistribution was further evaluated in different tissues by real time PCR of the recombinant viral genomes. Our results show that the pre-injection of polylysine resulted in a 5 to 12-fold increase of plasmatic mSeAP levels while the injection of polyinosinic acid resulted in a 2-fold increase of mSeAP expression. Interestingly, the polyinosinic acid pre-injection also resulted in a significant reduction of the neutralizing antibody titer raised against the AAV2 capsid.

## Materials and Methods

### Ethics Statement

Care and manipulation of mice were performed in accordance with national and European legislations on animal experimentation. The experiments were approved by the veterinary office of Essonne, France (Agreement number 91–89).

### Materials

Polyinosinic acid potassium salt, Poly-L-arginine hydrochloride (pArg) and Poly-L-lysine hydrobromide (pLys) of different molecular weights were obtained from Sigma (France). Polymer solutions for pre-injection were prepared extemporaneously by solubilization of the desired amount in 5% glucose.

### Recombinant AAV2 vector production

AAV2 vectors were produced in an adenovirus-free system by triple transfection using polyethylenimine (PEI 25 kDa; Sigma-Aldrich, France) as described previously [19]. Briefly, human embryonic kidney 293 cells were transfected in 15 cm plates with the trans-complementing adenovirus helper plasmid pXX6 [20], the pRepCap4 packaging plasmid expressing AAV2 *rep* and *cap* genes, and a cis acting AAV vector plasmid (pXL3937-CMV-mSeAP, pXL3937-MCK-mSeAP, pGG2-CMV-Luc or pGG2-CMV-LacZ). The AAV vector plasmids pXL3937-CMV-mSeAP, pGG2-CMV-Luc and pGG2-CMV-LacZ contain, respectively, the cDNA of a murine secreted alkaline phosphatase (mSeAP) [21], the luciferase gene and the cytosolic LacZ bacterial gene subcloned into an AAV plasmid backbone which has the ITRs of AAV2. The resulting AAV vector plasmids are under the transcriptional control of the cytomegalovirus immediate-early (CMV IE) promoter and the SV40 polyA sequence. The CMV promoter was replaced with a functional truncated muscle creatine kinase (MCK) promoter (CK6; [22]) in pXL3937-CMV-mSeAP to obtain pXL3937-MCK-mSeAP. After 72 h of transfection the cells underwent 4 cycles of freeze/thaw, the crude lysate was then treated with 25 U/ml benzonase, and AAV vectors were precipitated with cold saturated ammonium sulfate before purification by double CsCl_2_ ultracentrifugation gradient. This step was followed by extensive dialysis against sterile phosphate-buffered saline containing Ca^2+^ and Mg^2+^. The concentration of encapsidated viral genomes was determined by real-time quantitative PCR against a standard plasmid range and titers were expressed as viral genomes per ml (vg/ml). Titers were 7.3×10^11^ vg/ml for AAV2-CMV-mSeAP, 3×10^11^ vg/ml for AAV2-MCK-mSeAP, 2.4×10^11^ vg/ml for AAV2-Luc and 4.5×10^11^ vg/ml for AAV2-LacZ.

### *In vivo* experiments

Eight to ten week-old female Balb/C mice (Charles River, France) were injected in the tail vein with 200 µl of polymer solution 5 minutes before a second tail vein injection of the vector solution containing 1×10^11^ vg (unless otherwise stated) of the AAV in a final volume of 450 µl of sterile phosphate-buffered saline supplemented with Ca^2+^ and Mg^2+^. Co-injection consisted in a single tail vein injection of 650 µl of the mix of the polymer and AAV solutions. Retro-orbital plexus blood samples were obtained from anaesthetized mice the day before the injection and thereafter every week using heparinized capillary tubes. Plasma was obtained by centrifugation at 4000 g for 15 min and either analysed immediately or stored at −20°C. Mice were sacrificed by cervical dislocation. Several tissues were collected and frozen in liquid nitrogen-cooled isopentane.

### Histological analysis

*In situ* stainings were performed on 8 µm muscle transversal cryosections. The mSeAP and β-galactosidase detections on muscle sections were performed on slices fixed with 0.5% glutaraldehyde. For mSeAP detection, fixed slices were washed twice with PBS and endogenous alkaline phosphatase was heat-inactivated for 30 min at 65°C before overnight incubation at 37°C in 0.165 mg/ml 5-bromo-4-chloro-3-indolylphosphate (BCIP) and 0.33 mg/ml of nitroblue tetrazolium in 100 mM Tris-HCl, 100 mM NaCl and 50 mM MgCl_2_. For β-galactosidase detection fixed slices were washed twice with PBS containing 1 mM MgCl_2_ and incubated in 1 mg X-Gal/ml (5-bromo-4-chloro-3-indolyl-β-D-galactopyranoside), 5 mM K_3_Fe(CN)_6_, 5 mM K_4_Fe(CN)_6_, and 1 mM MgCl_2_ in PBS for 5 hours. mSeAP or β-galactosidase revealed sections were finally counterstained with nuclear fast red, mounted and analysed by light microscopy. Non-overlapping tiled images of complete transverse muscle sections were obtained using Cartograph software (Microvision, France) and a Nikon ECLIPSE E600 microscope with a Sony 3CCD DSP camera system using a 4× magnification objective.

### Blood and tissue analysis

mSeAP levels in plasma and in tissue extracts were quantified by chemiluminescent detection of the enzyme activity. Briefly, endogenous alkaline phosphatase was heat-inactivated for 5 or 30 min at 65°C (for plasma and tissue, respectively) and the heat-resistant mSeAP was quantified by addition of the reaction buffer and CSPD chemiluminescent substrate, according to the manufacturer's instructions (Phosphalight kit TROPIX, Applera). Chemiluminescence was measured in 96-well plate format with a luminometer (Perkin Elmer, Victor^2^ 1420 Multilabel counter). Expression levels were determined using a standard curve of purified human placental alkaline phosphatase and expressed as ng of mSeAP per ml of plasma. Tissues were quantified for protein content by fluorimetry using NanoOrange kit (Molecular Probes) to normalize mSeAP expression, and results were expressed as ng of mSeAP per mg of total protein in the extract.

### DNA isolation and real-time PCR

DNA isolation from tissues was performed using the Wizard Genomic DNA Purification Kit (Promega, France) in accordance with the manufacturer's protocol. Total DNA concentration was determined using a Nanodrop ND-8000 spectrophotometer (Nanodrop Technologies, France), and 70 ng of DNA of each sample was used as the template material for real-time-PCR. Taqman real-time PCR was performed on each sample for both the CMV promoter in order to determine copies of the viral genome, and the mouse titin gene, to standardize for number of mouse genomes present in each sample. Primers and probe used for CMV amplification were: 5′-CATCAATGGGCGTGGATAGC-3′ (forward), 5′-GGAGTTGTTACGACATTTTGGAAA-3′ (reverse) and 5′-ATTTCCAAGTCTCCACCC-3′ (probe). Primers and probe used for titin were: 5′-AAAACGAGCAGTGACGTGAGC-3′ (forward), 5′-TTCAGTCATGCTGCTAGCGC-3′ (reverse), and 5′-TGCACGGAAGCGTCTCGTCTCAGTC-3′ (probe). The PCR amplifications were performed using 70 ng of DNA diluted in Absolute QPCR ROX Mix (Thermo Fischer scientific, France), 0.1 µM of Taqman probes, and 0.2 µM primers (forward and reverse) in a final volume of 18 µl. Cycling conditions consisted of a Thermo-Start DNA Polymerase activation step at 95°C for 15 min followed by 40 cycles of two steps, 15 s of denaturation at 95°C and 60 s of annealing and extension at 60°C. The PCR was performed on a 7900 HT thermocycler (Applied Biosystem, France). A standard dilution range of a plasmid containing CMV and titin sequences was used on each real-time-PCR plate as copy number control. All samples and controls were run in duplicate. Data are expressed as the number of viral genome copies per diploid genome (copies/nucleus).

### Neutralization assay

On day 1, 48-well plates were seeded with 5×10^4^ Hela cells/well and incubated for 24 h. On day 2, recombinant AAV2-CMV-Luc was diluted in DMEM (Invitrogen Life Technology, Auckland, CA, USA) supplemented with 10% fetal calf serum and incubated with a 10-fold dilution, then 2-fold serial dilutions (1∶20 to 1∶12800) of heat inactivated (at 56°C for 30 min) plasma samples for 1 h at 37°C. Subsequently, the plasma-AAV2-CMV-Luc vector mixtures corresponding to 5×10^3^ viral genome/cell, were added to cells plated on day 1 and incubated in DMEM +10% FCS for 48 h at 37°C and 5% CO_2_. Each mix was performed in duplicate. Cells were then washed in PBS and lysed for 10 minutes in 0.2% Triton Lysis Buffer at 4°C. The lysate was transferred to 96-well plates then the luciferase activity was read on a luminometer (VICTOR^2^ 1420 multilabel counter, Perkin Elmer/Life Sciences). Transduction efficiency was measured as relative light units (RLU), per second per well and normalized per amount of protein per well expressed as optical density (RLU/sec/w/OD). The percentage of transduction inhibition is then calculated for each plasma dilution relatively to the maximal transduction efficiency which is determined by a control AAV infection in presence of PBS. We added sera from 5 mice before injection as negative control and we checked the absence of pI interference in the neutralizing assay using sera from mice injected with pI alone at 200 µg/mouse. The neutralizing antibody factors (NAF) were defined as the reciprocal plasma dilution inhibiting maximal transduction by 50%.

### Statistical analysis

All data are expressed as means +/− standard deviation (SD). Differences between two groups were tested by using the unpaired T test with Welch correction as SDs could be different between the two populations tested. Statistical significance was defined by a two-tail *p* value below 0.05.

## Results

### Effect of pre-injection of cationic polymers on the AAV2 transduction efficiency

To monitor gene expression, we used as reporter gene a murine secreted alkaline phosphatase (mSeAP). mSeAP expression results in the production of a non immunogenic protein which allows specific detection in blood and tissues. Notably, the background is very low due to a higher thermostability of mSeAP as compared to the endogenous phosphatases [21].

**Figure 1:**
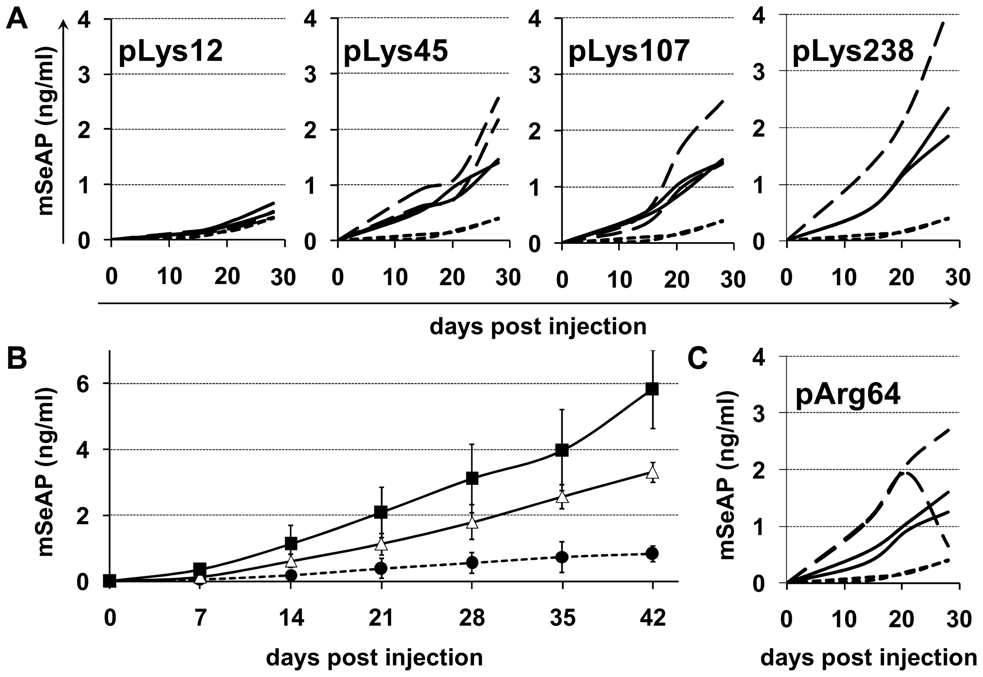
Kinetic of mSeAP secretion following the pre-injection of various cationic polymers. The mSeAP secretion in blood was measured after pre-injection of pArg64 or with pLys of different degrees of polymerization followed five minutes later by an intravenous injection of 1×10^11^ vg of AAV2-CMV-mSeAP. Plasma concentration of mSeAP was monitored every week until day 28 (**A** and **C**) or day 42 (**B**). (**A**) Mice were injected with AAV alone (dotted line), or pre-injected with 120 µg (dashed lines) or 150 µg (solid line) of pLys before AAV2 infusion with n = 2 animals per condition (each curve represents a mouse). Notably, one mouse died in the group injected with 150 µg pLys238. (**B**) Comparison of mSeAP secretion in blood after a pre-injection of pLys45 or pLys238 followed five minutes later by an intravenous injection of 1×10^11^ vg of AAV2-CMV-mSeAP. Mice were injected with AAV alone (dotted line and filled circles; n = 20), or pre-injected with 150 µg of pLys45 (triangles; n = 9) or 150 µg of pLys238 (squares; n = 14) before AAV2 infusion. (**C**) Mice were injected with AAV alone (dotted line), or pre-injected with 120 µg (dashed lines) or 150 µg (solid line) of pArg64 before AAV2 infusion (n = 2 and each curve represents a mouse). Results are expressed as the average value +/− SD.

As mentioned in the introduction, we hypothesized that pre-injection of cationic polymers could increase the transduction efficiency of AAV2. Since the size of the polymer may play a role, we evaluated four different poly-L-lysines (pLys) with increasing degrees of polymerization (dp): pLys12, pLys45, pLys107, and pLys238. The polymers diluted in 200 µl of a 5% glucose solution were injected 5 minutes prior administration of 450 µl containing 1×10^11^ vg of AAV2. The kinetics of the secretion of the reporter protein was followed over a period of 28 days using the blood samples which were taken once per week. The results indicate a critical effect of the size of the polymer. Indeed, while pLys12 did not alter the mSeAP levels at any time point as compared to the non pre-treated AAV2 group, the 3 pLys with higher degrees of polymerization enhanced the expression of reporter gene at all time points (Figure 1A). In this first experiment, the pLys of dp 238 displayed some toxicity since one of the 2 mice died after the 150 µg injection. Further experiments indicated that pLys238 was lethal in 50% of cases after injection of 175 to 200 µg. However, at the doses used to perform the study (i.e. 150 µg) mortality never exceeded 20% of injected mice (not shown).

As shown in Figure 1A, the pre-injection of pLys238 resulted in a higher transgene secretion than when using for example the pLys with dp of 45. To confirm this point, we repeated the experiment with these two polymers. Figure 1B shows that there is a significant difference between both polymers. The enhancement factor of mSeAP secretion was of 4 and 7 at day 42 after pre-injection of pLys45 and pLys238, respectively (statistical difference was achieved between pLys45 and pLys238 at every mean point of the kinetic). Taking into account this result, we used pLys238 in the subsequent experiments.

In order to check whether this enhancement effect on AAV *in vivo* efficiency is restricted to polylysines, we pre-injected another cationic polymer before the infusion of AAV2: a poly-arginine with a dp of 64 (pArg64). As shown in Figure 1C, the pre-treatment with pArg64 allowed for an increase of the secretion of mSeAP of the same order than the polylysines. This suggests that the pre-injection of a cationic polymer displays a general enhancing effect on AAV-mediated gene delivery.

Next, we tested the influence of the time-lapse between cationic polymer pre-injection and AAV administration. The results indicated that while co-injection of both, polymer and AAV, resulted in similar effects than with a 5 minute pre-injection, we also found that longer periods between the two injections reduced the effect (data not shown).

**Figure 2:**
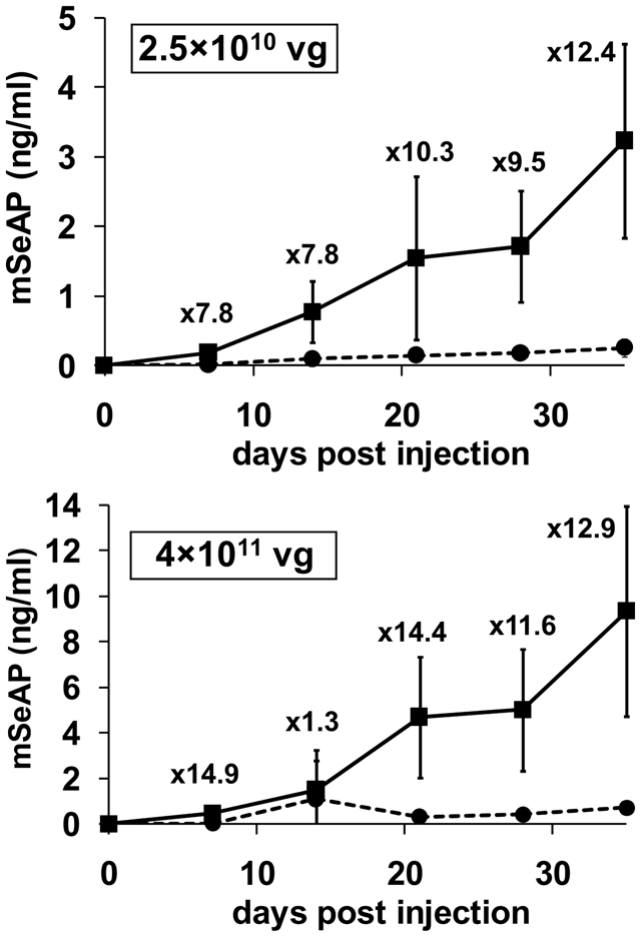
Effect of pLys238 using two AAV2 vector doses. The mSeAP secretion in blood was measured after pre-injection of pLys238 followed five minutes later by an intravenous injection of either 2.5×10^10^ or 4×10^11^ vg of AAV2-CMV-mSeAP. Plasma concentration of mSeAP was monitored every week for 5 weeks. Mice were injected with AAV2 alone (dotted line and filled circles), or pre-injected with 150 µg of pLys238 (squares) before AAV2 infusion. Results are expressed as the average value +/− SD, with n = 3 in group 2.5×10^10^ and n = 4 in group 4×10^11^ vg. The fold increase in mSeAP levels induced by the pLys238 treatment is indicated for each time point.

In order to check whether the enhancement mediated by pLys is AAV2 dose-dependent, we pre-injected 150 µg of pLys238 before infusion of either 2.5×10^10^ or 4×10^11^ vg. As shown in Figure 2, the pre-injection of the cationic polymer resulted in an increase of the mSeAP levels in blood of about a factor 12. These results clearly show that the pLys effect is AAV2-dose independent.

**Figure 3:**
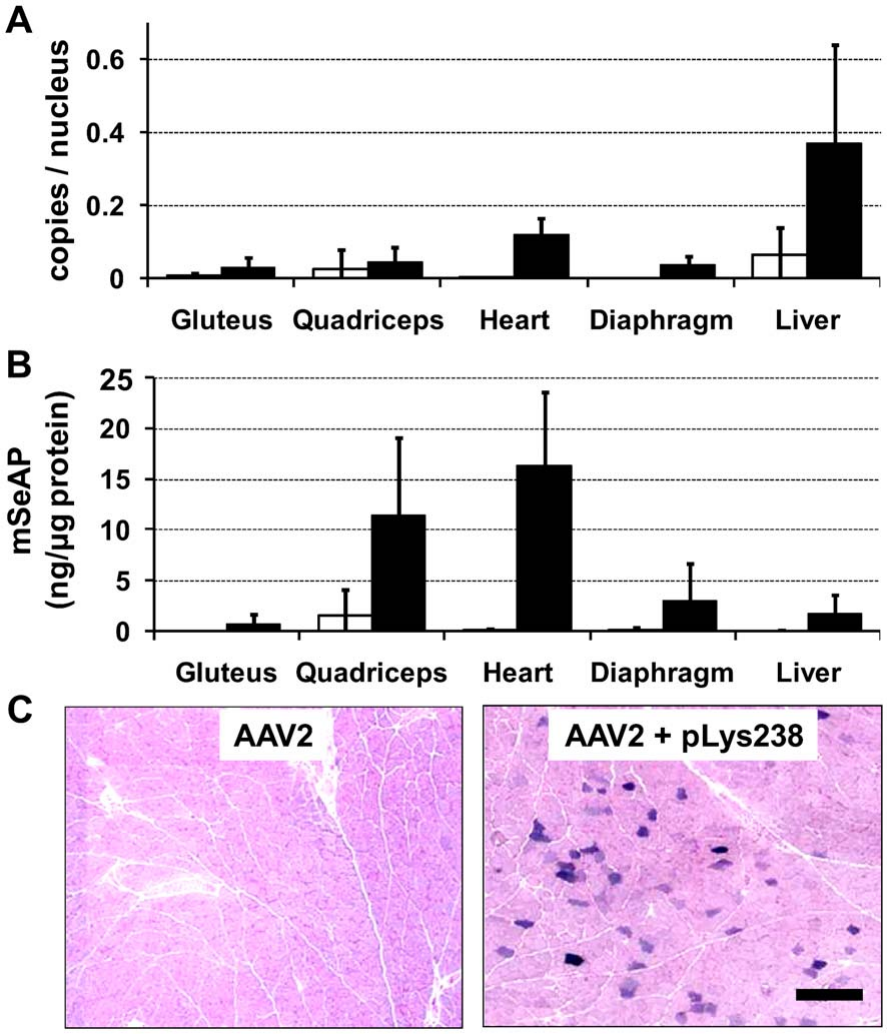
AAV2 tissue transduction and biodistribution following pLys238 pre-injection. (**A**) The vector tissue biodistribution in mice injected with 4×10^11^ vg of AAV2-CMV-mSeAP with (black bars) or without (white bars) the pre-injection of 150 µg pLys238 was analysed by real time PCR in genomic DNA extracted from different muscles and from the liver. (**B**) Tissue lysates were also subjected to the quantification of mSeAP. For (**A**) and (**B**) results are expressed as the average value + SD, with n = 4 in each experimental group. (**C**) *In situ* histochemical staining of mSeAP in quadriceps muscles. Quadriceps muscles were collected on day 35 after injections. mSeAP was revealed using the NBT/BCIP method and counterstained with nuclear fast red. A representative image of mice injected with the AAV2 alone is presented on the left, and the right panel corresponds to mice pre-injected with 150 µg of pLys238 before AAV2 infusion. Scale bar represent 200 µm.

The mice that received 4×10^11^ vg of AAV2 with or without a pLys pre-injection were analyzed in greater details. First, by using quantitative real-time PCR, we determined the copy number of viral genomes present in different tissues. As revealed by the results (Figure 3A) the pre-injection of the cationic polymer increased the copy number in all analyzed tissues, the greatest increase being found in the heart, diaphragm and liver (30-, 15- and 5-fold increase, respectively as compared to the AAV2 treated group). To check whether there is a correlation between copy number of viral genomes and the expression level we quantified the amount of mSeAP present in the tissue lysate. The mSeAP quantification in the various tissues showed a good correlation with the Q-PCR results, except for the liver where the expression levels were particularly low considering that it was the tissue with the highest vg copy number (Figure 3B). This can be explained by the well-documented fact that liver is prone to CMV promoter extinction [23], [24]. Since mSeAP can also be revealed on tissue sections, we looked whether and to which extent skeletal muscle was transduced. As shown in Figure 3C, while no positive fibers could be seen in the quadriceps of mice from the AAV2 group, a significant amount of fibers were mSeAP positive in the AAV2 treated mice pre-injected with pLys238. Altogether, the data from Q-PCR, mSeAP quantification from tissue lysate and histochemistry of tissue section are in good agreement and show that pLys increases the overall transduction of all tested tissues.

**Figure 4:**
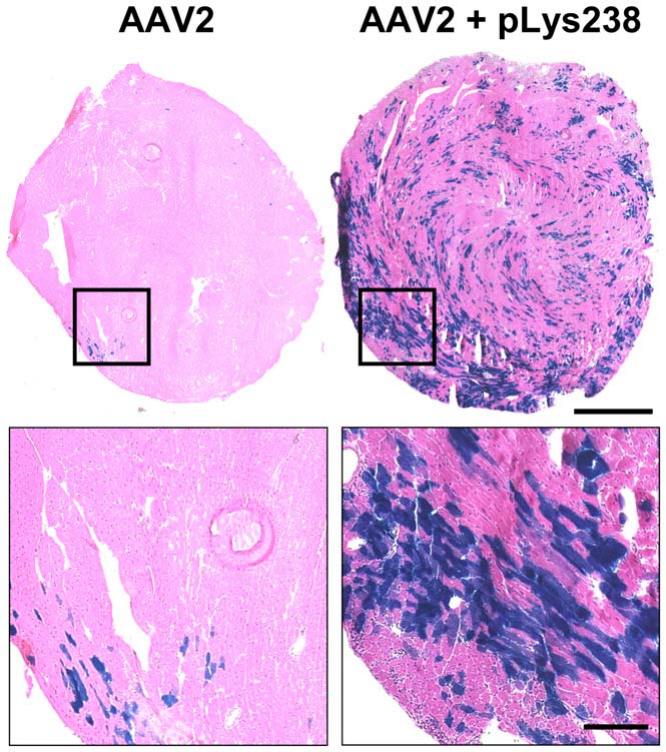
Intravenous delivery of AAV2-LacZ following pLys238 injection results in high-level transduction of the heart. X-gal staining of cross-sections of hearts after systemic administration of 2×10^11^ vg of AAV2-CMV-LacZ. The best results obtained with both conditions (AAV2 +/− pLys) are represented here: the photography on the top left corresponds to a mouse injected with the vector alone, and the photography on the top right corresponds to a mouse pre-injected with 150 µg of pLys238 five minutes before AAV2 infusion. Hearts were collected on day 28 post injections, n = 3 in each experimental group. Complete sections are presented on the top (1 mm scale bar); black boxes at the top are seen at a higher magnification below (200 µm scale bar).

Notably, *in vitro* data from a transfected cell culture showed that about 90% of mSeAP is secreted, while the remaining 10% are found in the cell lysate [21]. As most of the mSeAP is secreted, we probably underestimate the amount of transduced cells in tissues, especially when using low vector doses. To circumvent this problem, we made use of an AAV2 vector encoding LacZ. Due to the fact that we used a non-secreted cytoplasmic form of this reporter gene, we expected a higher sensitivity as compared to mSeAP. The results showed again a major improvement of heart transduction as seen in Figure 4. Indeed, very few cardiomyocytes expressed β-galactosidase in control mice injected with AAV2 alone whereas pre-injection of pLys238 enabled AAV2 to transduce a large amount of cardiomyocytes.

**Figure 5:**
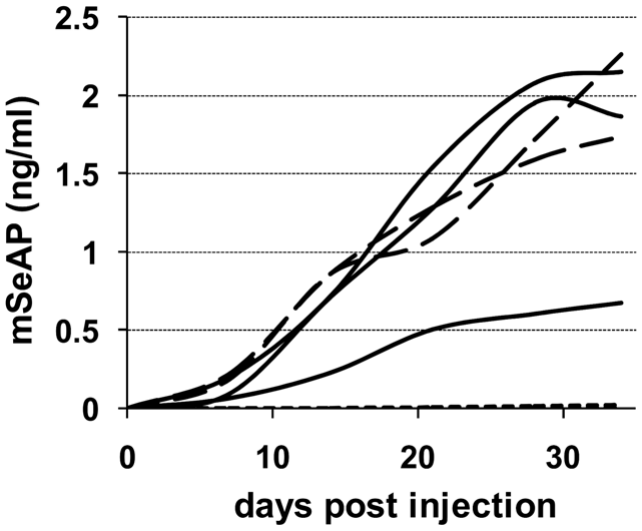
Muscle-specific mSeAP secretion following injection of AAV2-MCK-mSeAP +/− pLys. The mSeAP secretion in blood was measured after pre-injection of 125 µg pLys45 (solid lines) or 150 µg of pLys238 (dashed lines) followed five minutes later by an intravenous injection of 1×10^11^ vg of AAV2-MCK-mSeAP. Plasma concentration of mSeAP was monitored every week for 5 weeks. Mice injected with the vector alone (dotted lines; close proximity to the X axis) are also shown (n = 4). Each curve represents a mouse.

It has been previously shown that tail vein injection in mice of an AAV vector coding for a reporter gene under the control of the muscle creatine kinase (MCK) promoter results in strong expression in muscles while expression in nonmuscle tissues remains ≤4% of the highest activity seen in most skeletal muscles [22]. We used the same MCK promoter (AAV2-MCK-mSeAP) in order to follow more specifically the effect of polylysine pre-injection on the transduction of skeletal muscles. Our results show that while in the absence of injection of pLys the plasma level of mSeAP was extremely low (11 pg/ml on day 34), a pre-treatment with either pLys45 or pLys238 dramatically increased the reporter gene expression (Figure 5). This experiment clearly demonstrates that pre-injection of a cationic polymer improves muscle transduction.

### Effect of pre-injection of polyinosinic acid on AAV2 mediated *in vivo* transduction

**Figure 6:**
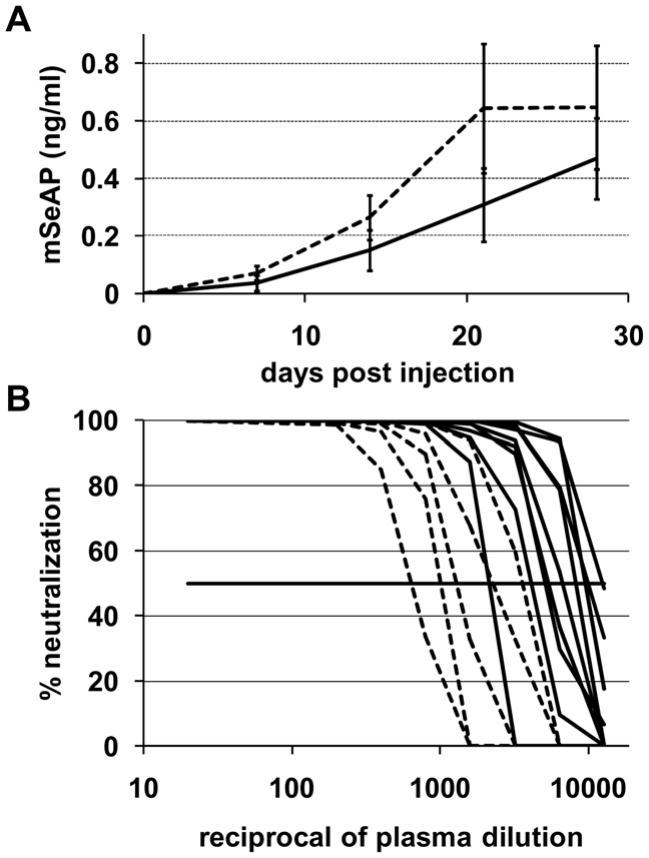
Impact of polyinosinic acid pre-injection on the transduction efficiency and antibody response against AAV2. (**A**) The mSeAP secretion in blood was measured after the pre-injection of 200 µg pI followed five minutes later by an IV injection of 1×10^11^ vg of AAV2-CMV-mSeAP. Plasma concentration of mSeAP was monitored every week for 4 weeks. Results are expressed as the average value +/− SD, with n = 9 for mice injected with the vector alone (solid line), and n = 5 for mice treated with pI (dotted line). (**B**) Inhibition of neutralizing factors (NAF) generated against AAV2 with polyinosinic acid pre-injection. NAF from plasma collected at day 28 were titrated. Serial dilutions of plasma from mice injected with AAV2 with or without a pre-injection of 200 µg pI were incubated with the identical serotype-based AAV2-Luc encoding the luciferase gene. Residual AAV infectivity was then measured on HeLa cells. Each curve represents a mouse: dotted curves represent pI treated mice and solid curves represent control mice injected with the vector alone.

Based on the results obtained by other groups with recombinant adenoviruses [13], we asked whether the pre-injection of an anionic polymer such as polyinosinic acid (pI) could increase the transduction efficiency of AAV2. A putative mechanism could be, for example, a polyinosinic acid saturation of the scavenger receptors of Kupffer cells, and this in turn could lead to a decreased viral uptake. We injected 1×10^11^ vg of AAV2-CMV-mSeAP 5 minutes after infusion of 200 µg of pI. The plasma level of mSeAP was determined at different time points. As shown in Figure 6A, the pre-treatment led to a low but significant increase of the mSeAP plasma level.

Since pI may have reduced, or inhibited, the transduction of antigen presenting cells (APCs) by binding to scavenger receptors, we asked whether such a treatment could reduce the humoral response directed against AAV2 capsid proteins. We quantified the neutralizing factors (NAF) in mice 28 days after injection. Neutralizing antibodies from injected mice were titrated *in vitro* by a virus neutralization assay, using the plasma from the animals (Figure 6B). Results indicate that the pI pre-treatment resulted, in average, in a significant 4-fold lower NAF titer than the control group (1/1800 vs 1/7400 mean titer). This result clearly suggests that intravenous injection of pI before infusion of AAV2 particles results in a reduced uptake of the virus by APCs, which in turn leads to a reduced production of NAF.

Notably, we also quantified the NAF in 150 µg pLys238-treated mice. The results showed that this latter treatment did not change the NAF titer as compared to the AAV2 injected group (Figure S1). This suggests that pLys238 does not increase the transduction of professional antigen presenting cells.

## Discussion

The success of gene therapy for the treatment of genetic diseases such as muscular dystrophies requires widespread and stable gene delivery. Adeno-associated viruses (AAV) are, to date, the only vectors that enable body-wide gene delivery. However, this can only be achieved by injecting extremely high amounts of viral particles. For example, using AAV-6, a serotype which efficiently transduces muscle, 10^12^ vg (about 5×10^13^ vg/kg) have to be administered for widespread transduction of both cardiac and skeletal muscles in adult mice [1]. Based on this and other results, it is hypothesized that ≥10^15^ vg will have to be administered intravenously to obtain similar results in humans. Besides raising safety questions, the feasibility of producing such large amounts of AAVs in GMP conditions remains to be shown. Indeed, with the routine method consisting in the transfection of HEK293 cells cultured in 15 cm diameter plates, production of 10^15^ particles would require 5700 plates [25].

Here, we investigated whether polylysine or polyinosinic acid could act as adjuvants and improve the *in vivo* transduction efficiency. The results obtained with cationic polymers, and in particular polylysines, showed that co- or pre-injection a few minutes before infusion of AAV2 particles significantly increases the overall transduction efficiency. This was shown by quantitative PCR, as well as by the mSeAP expression in plasma and in different tissues. However, it is to be noted that the increase was not similar in all tissues: the greatest effects were obtained in the heart and diaphragm. The magnitude of the improved transduction was, depending on the experiment, 5 to 12-fold more mSeAP than in the control group. Notably, this effect was not AAV2 dose-dependent. Interestingly, the enhancement of the mSeAP secretion was correlated to the polymerization degree of the pLys. This suggests that a minimal size of the polymer is required for boosting the transduction. The results obtained after the injection of pArg64 (Figure 1C) show that it is also possible to increase the transduction efficiency of AAV2 by using other cationic polymers than polylysines. This finding is important since it means that alternative compounds which may have less toxic effects than pLys238 exist. The exact reasons for the lethality provoked by injection of high doses of pLys238 are unknown. Of note however, our results are in agreement with the study of Arnold and colleagues which showed that *in vivo* toxicity of polylysines decreases with the molecular weight of the polymer [26]. These authors suggest that cytotoxicity of polylysines results from membrane perturbations which, for example, affect membrane permeability to small molecules. Besides the intrinsic toxicity of high doses of pLys238, one could mention two other parameters that may have played a role in the toxic effects: firstly, the purity of the polymer. Indeed, pLys238 is a mixture of polylysines (the molecular weight ranges between 30,000 and 70,000) and we can not exclude the presence of cytotoxic contaminants. Secondly, the counterion of pLys238 is bromide, a ion that has been shown to be toxic for cells [27]. Taken together, high molecular weight polylysines are cytotoxic at high doses but it may be possible to reduce this toxicity by using a highly purified polylysine and by exchanging the bromide ion by chloride for example. Alternatively, in view of clinical applications one could use polylysines of lower molecular weight which are less toxic or use other cationic polymers.

How exactly the polymers act to increase the transduction remains unknown. One possibility could be that the effect results from the modification of the surface charge of the AAV particles. Indeed, it has been reported that the surface charge (zeta-potential) of AAV2 is negative and that addition of a cationic compound such as protamine modifies its surface charge [28].

The results obtained with AAV2 encoding for mSeAP under the control of the muscle specific promoter MCK are interesting, since the increase after pLys treatment of the circulating mSeAP levels is impressive. It has been reported that the synthesis of heparan sulfate proteoglycan is down-regulated during murine skeletal muscle maturation, and this has been proposed to be responsible for the loss of HSV infectivity [29] (heparan sulfate acts as a co-receptor for attachment of HSV to cells). Based on this report, we propose the hypothesis that due to low levels of heparan sulfate proteoglycan in muscle, cationic polymers improve the transduction by facilitating the primary interaction between virus and the muscle fibers. Co-receptors would subsequently intervene for virus internalization. Alternatively, pLys could neutralize the anionic charges present on the vascular endothelium and this, in turn, could favour AAV2 tissue penetration.

Based on results obtained with adenoviruses [13], we evaluated a second strategy which consisted in pre-injecting the anionic polymer polyinosinic acid. In fact, we asked whether an injection of pI could prevent infection by AAV2 of Kupffer cells or other antigen presenting cells and thus, not only enhance the transduction of other tissues but also reduce the humoral immune response against AAV2 capsid proteins. The results showed that a pI treatment resulted in a two-fold increase of the circulating mSeAP levels. A second, interesting effect of the pre-treatment was the fact that it reduced by 4-fold the levels of neutralizing factors directed against the capsid proteins. We attribute this latter effect to an inhibition of AAV2 uptake by antigen presenting cells, due to the inhibition of scavenger receptors by pI. This is of particular importance when considering the fact that the immune response against AAV vectors after a first injection hinders future re-administrations. Although the present reduction in NAF may not be sufficient to allow a re-administration, it opens new perspectives for developing strategies that will be more efficient than a pI pre-administration.

In conclusion, our results show that pre-injection of polymers, especially cationic ones, have the potential to significantly increase the transduction efficiency of various tissues, including skeletal muscle. This strategy potentially allows a reduction of the injected vector dose as compared to an injection of AAV2 alone. This is not only a benefit in terms of safety (a lower viral load reduces potential for immune response to the capsid proteins) but it is also of real interest from a GMP production perspective. Indeed, if one can divide by several-fold the production scale this will represent a very significant reduction of the costs.

## Supporting Information

Figure S1 **Impact of pre-injection of pLys238 on the antibody response against AAV2.** Neutralizing factors (NAF) generated against the capsid proteins of the vector in AAV2 and pLys238/AAV2 treated animals were titrated in plasma collected at day 28. Serial dilutions of plasma from mice injected with 4×10^11^ vg of AAV2 (experiment Figure 2) with or without a pre-injection of 150 µg pLys238 were incubated with AAV2-CMV-Luc encoding the *luciferase* gene. Residual AAV2 infectivity was then measured on HeLa cells. Each curve represents a mouse: dotted curves represent pLys238 treated mice (n = 3) and solid curves represent control mice injected with the vector alone (n = 3).
(TIF)
