# Supplementary figures and images for: Polymers for Improving the In Vivo Transduction Efficiency of AAV2 Vectors
Source: PLoS One. 2010 Dec 28;5(12):e15576. doi: 10.1371/journal.pone.0015576 (PMC3011005; doi:10.1371/journal.pone.0015576)

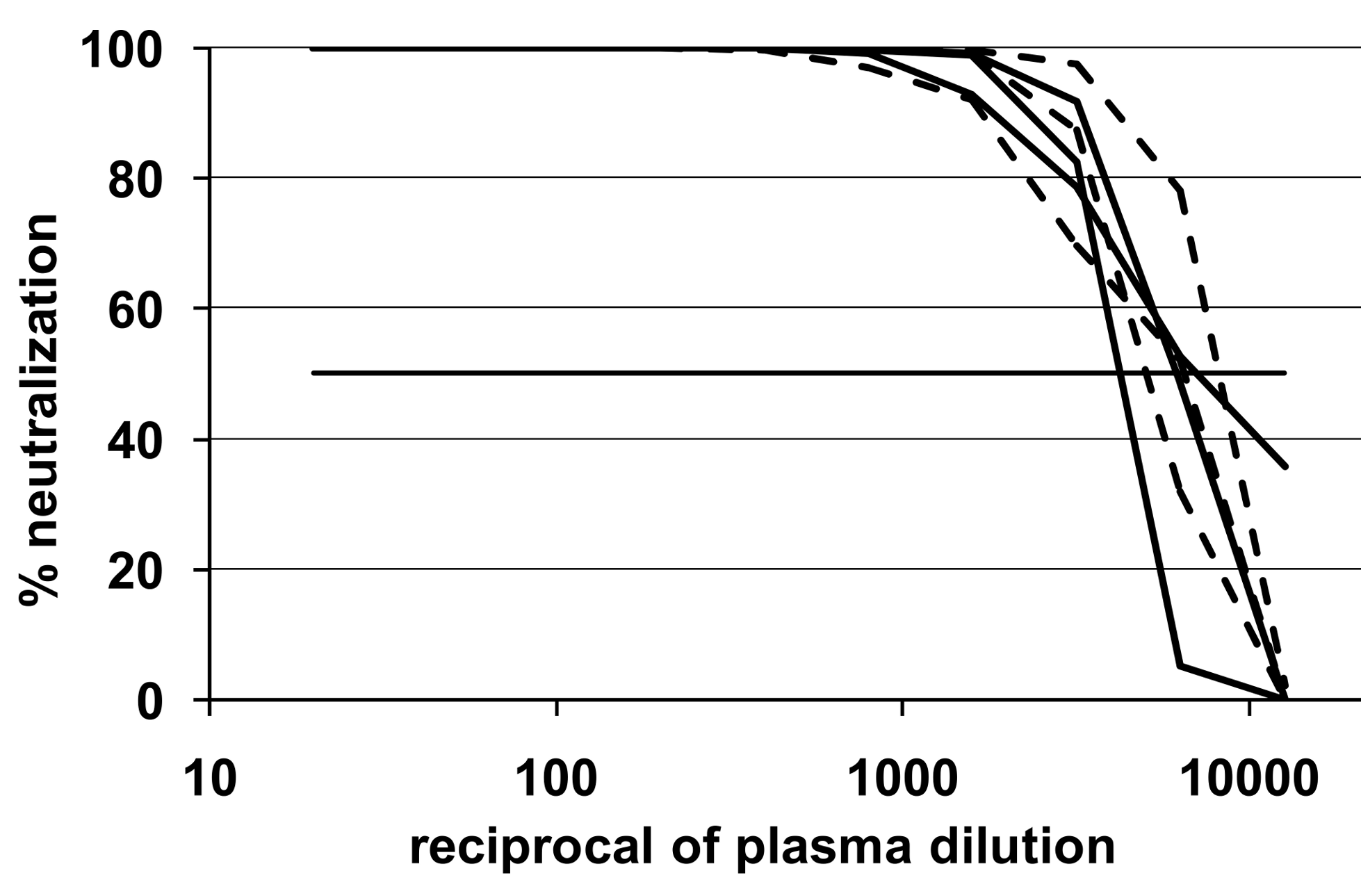

Supplement: Figure S1 — Impact of pre-injection of pLys238 on the antibody response against AAV2. Neutralizing factors (NAF) generated against the capsid proteins of the vector in AAV2 and pLys238/AAV2 treated animals were titrated in plasma collected at day 28. Serial dilutions of plasma from mice injected with 4×10^11^ vg of AAV2 (experiment Figure 2) with or without a pre-injection of 150 µg pLys238 were incubated with AAV2-CMV-Luc encoding the luciferase gene. Residual AAV2 infectivity was then measured on HeLa cells. Each curve represents a mouse: dotted curves represent pLys238 treated mice (n = 3) and solid curves represent control mice injected with the vector alone (n = 3). (TIF) [file pone.0015576.s001.tif]
